# Impact of time-of-flight on qualitative and quantitative analyses of myocardial perfusion PET studies using ^13^N-ammonia

**DOI:** 10.1007/s12350-014-0037-8

**Published:** 2014-12-03

**Authors:** Takeshi Tomiyama, Keiichi Ishihara, Masaya Suda, Koji Kanaya, Minoru Sakurai, Naoto Takahashi, Hitoshi Takano, Koichi Nitta, Kenta Hakozaki, Shin-ichiro Kumita

**Affiliations:** 1 Department of Radiology, Nippon Medical School, 1-1-5 Sendagi, Bunkyo-ku, Tokyo, 113-8603 Japan; 2 Clinical Imaging Center for Healthcare, Nippon Medical School, Tokyo, Japan; 3 Department of Cardiovascular Medicine, Nippon Medical School, Tokyo, Japan; 4 NM & RT System Sales Division, Hitachi Medical Corporation, Tokyo, Japan

**Keywords:** ^13^N-ammonia, Time-of-flight (TOF), Myocardial blood flow (MBF), Myocardial flow reserve (MFR), Positron emission tomography (PET)

## Abstract

**Background:**

The impact of time-of-flight (TOF) in myocardial perfusion ^13^N-ammonia positron emission tomography (PET) is unclear.

**Methods and Results:**

Twenty consecutive subjects underwent rest and adenosine stress ^13^N-ammonia myocardial perfusion PET. Two sets of images were reconstructed using TOF-ordered subset expectation maximization (TOF-OSEM) and 3-dimensional row-action maximum likelihood algorithm (3D-RAMLA). Qualitative and quantitative analyses from the TOF-OSEM and 3D-RAMLA reconstructions were compared. Count profile curves revealed that TOF relatively increased the uptake of ^13^N-ammonia at the lateral walls, and apical thinning was emphasized on the TOF images. Both segmental rest and stress myocardial blood flow (MBF) values were higher with TOF-OSEM use than with 3D-RAMLA use (rest MBF: 0.955 ± 0.201 vs 0.836 ± 0.185, *P* < .001; stress MBF: 2.149 ± 0.697 vs 2.058 ± 0.721, *P* < .001). The differentiation of MBF between reconstructions was more enhanced under rest conditions. Thus, segmental myocardial flow reserve (MFR) observed using TOF-OSEM reconstruction was lower than that observed using 3D-RAMLA (2.25 ± 0.57 vs 2.46 ± 0.75, *P* < .001). No remarkable differences were observed between segmental and territorial results.

**Conclusions:**

TOF increased lateral wall counts and emphasized apical thinning. Quantitatively, TOF reconstruction showed increased MBF, especially under relatively low perfusion conditions.

## Introduction

^13^N-ammonia myocardial perfusion positron emission tomography (PET) can quantitatively evaluate the absolute rest and stress myocardial blood flow (MBF) and myocardial flow reserve (MFR). As coronary stenosis increases in severity, both stress MBF and MFR reduce.^1^ MFR changes also occur because of microvascular and vascular endothelial dysfunctions caused by hypertension, diabetes mellitus, dyslipidemia, or smoking.^2^-^5^ Quantitative analysis in addition to a visual assessment improves the detection of chronic coronary artery disease (CAD).^6^ Furthermore, absolute MBF is useful for stratifying the risk of major adverse cardiac events and cardiac death, and predicting a long-term prognosis.^7^-^9^ ^13^N-ammonia PET can provide excellent images with few artifacts. However, because accumulation of ^13^N-ammonia in the lateral wall is about 10% lower than that in other segments even in healthy subjects, special attention is needed during interpretation.^10^

Time-of-flight (TOF)-PET/CT scans can measure the time difference between the detection of two 511 keV annihilation photons. TOF focuses on localizing events along coincidence line-of-response and improves the signal-to-noise ratio. An improvement in the signal-to-noise ratio and detectability has also been reported with whole-body oncologic PET.^11^,^12^ However, the impact of TOF on myocardial perfusion and quantification of MBF with ^13^N-ammonia PET has not yet been investigated. As such, the aim of this study was to assess the effect of TOF on qualitative and quantitative analyses of myocardial perfusion ^13^N-ammonia PET.

## Methods

### Study Population

Total 20 consecutive subjects underwent rest and stress ^13^N-ammonia myocardial perfusion PET. The baseline characteristics of the subjects are summarized in Table 1. Seven subjects were normal volunteers (3 male, 4 female; mean age ± SD, 52.0 ± 9.6 years; age range, 32 to 65 years) who were in good health and had never undergone coronary CT angiography or coronary angiography (CAG); some of the subjects had hypertension, dyslipidemia, or diabetes mellitus. Thirteen patients (8 male, 5 female; mean age ± SD, 69.5 ± 7.9 years; age range 54 to 81 years) had known coronary stenosis. Coronary stenosis was defined as >50% diameter stenosis shown by CAG. Written informed consent was acquired in all subjects. This study was approved by the local ethics committee and performed in accordance with the ethical standards of the Declaration of Helsinki.

**Table 1:** Baseline characteristics

Variable	(N = 20)
Age (years)	63.4 ± 11.9
Female	9
Coronary stenosis, no of vessels	
None (volunteers)	7
1	3
2	5
3	5
Hypertension	12
Diabetes mellitus	6
Dyslipidemia	14
Smoking	
Past smoker	6
Present smoker	1
Obesity (BMI ≥25 kg/m^2^)	7
Prior CABG	0
Prior PCI	6

*CABG*, coronary artery bypass grafting; *PCI*, percutaneous coronary intervention

### Data Acquisition

All subjects had totally fasted for >6 hours and refrained from caffeine-containing beverages and food for 24 hours prior to imaging. No subjects used theophylline-containing medications. PET was performed on a GEMINI TF-16 (Philips Medical Systems), which is a hybrid TOF-PET/CT scanner, under the following specifications: 28,336 lutetium-yttrium-oxyortho-silicate (LYSO) (4 × 4 × 22 mm^3^ in size), an 11.5% energy resolution, a timing resolution of 575 ps, and a localization accuracy of 8.62 cm.

Data acquisition was performed in the three-dimensional list mode. The scatter correction was a single scatter simulation algorithm, while the attenuation correction was based on rest and stress cardiac CT scans. During both CT and PET acquisitions, subjects breathed normally, but refrained from deep breathing, speaking, and body motion to avoid misregistration between CT and PET images. At rest, all subjects were injected ~370 MBq of ^13^N-ammonia into a peripheral vein, irrespective of subjects’ weight, followed by a 30 ml saline flush. Dynamic imaging was begun just before injection and extended for 10 min. Fifty minutes later (after decay), adenosine stress test was performed at 120 µg/kg/min for 6 min, according to Japanese clinical conventions. And then, at the end of the 3 min of infusion, ^13^N-ammonia was injected. Heart rate, blood pressure, and a 12-lead electrocardiogram (ECG) were recorded every minute during and after adenosine infusion, with continuous ECG monitoring.

### PET Image Reconstruction and Processing

Twenty-eight dynamic frames were reconstructed (24 × 5 second, 2 × 30 second, 1 × 1 minute, and 1 × 6 minute, for a total of 10 min). From the same raw data, two sets of images were reconstructed using TOF-ordered subset expectation maximization (TOF-OSEM) and 3-dimensional row-action maximum likelihood algorithm (3D-RAMLA).^13^,^14^ The 3D-RAMLA reconstruction, which is considered a special case of OSEM requiring sequences of orthogonal projections and a relaxation parameter to control updating of the log-likelihood objective at each full iteration cycle, was used as a non-TOF method.^15^ The reconstruction parameters for TOF-OSEM were set at three iterations and 33 subsets, whereas for 3D-RAMLA, there were two iterations. The relaxation parameters (*λ*) were 1.0 for TOF-OSEM and 0.08 for 3D-RAMLA. These parameters were optimized for the clinical application of dynamic reconstructions using vendor’s default.

### Visual Assessment

From the same raw data, TOF-OSEM and 3D-RAMLA images were re-sliced in the short axis, as well as in the vertical and horizontal long-axis orientations. Regional ^13^N-ammonia uptake was assessed using the AHA 17-segment model, and the semi-quantitative scoring system of defect severity and extent.^16^ A well-accepted 5-point scale scoring by two experts was carried out, as follows: 0, no defect; 1, mildly reduced; 2, moderately reduced; 3, severely reduced; and 4, absent of activity. For 13 CAD patients, diagnostic performance was compared between reconstructions based on CAG results (>50% stenosis).

### Count Profile Analysis

For the count profile analyses, 7 normal volunteers and 3 patients having normal perfusion were included. For each subject, 16 count profile curves were created: apical, middle, and basal levels of the short axis and the vertical long-axis images from both TOF-OSEM and 3D-RAMLA, under rest and adenosine stress conditions. Three lines were drawn at the short axis images from the septal to lateral walls, while lines across the apex were drawn at the vertical long-axis images. The lateral-to-septal ratio was acquired by dividing the lateral counts by the septal counts. The apex-to-mid ratio was determined as follows: apex/([mid septal + mid lateral]/2). All data were adopted by maximum counts. The lateral-to-septal ratios of the three levels of the short axis images, and the apex-to-mid ratio were compared between TOF-OSEM and 3D-RAMLA images. Additionally, the contrast between the myocardium (septal and lateral walls) and the left ventricular blood-pool was calculated from the count profile curves at the mid-level of the short axis, as follows:

$$ {\text{Contrast}} = \left( {I_{\hbox{max} } {-}I_{\hbox{min} } } \right)/\left( {I_{\hbox{max} } + I_{\hbox{min} } } \right), $$

where *I*_max_ is the maximum count of the septal or lateral wall, *I*_min_ is the minimum count of the left ventricular blood-pool.

### Absolute Quantification of Myocardial Blood Flow

Quantitative analyses were performed using PMOD software package (version 3.4, PMOD Technologies Ltd., Zurich, Switzerland). Regions of interest were semi-automatically placed on the myocardium and the biventricular blood-pool in each slice. Myocardial and blood-pool time-activity curves, generated from the dynamic frames, were fitted with the tracer kinetic model. We adopted the DeGrado 1-compartment model which assumes that there is no metabolic trapping.^17^ The first 4 min (27 frames) data were used for the curve fitting. The estimated rest and stress MBF were expressed in each segment and territory, while the MFR was calculated as the stress-to-rest MBF ratio. Normal ranges of values have been previously reported^18^; the rest and stress MBF ranged from 0.8 ml/min/g to 1.2 ml/min/g and from 2.7 ml/min/g to 4.6 ml/min/g of tissue, respectively. Accordingly, the MFR ranged from 2.9 to 4.4. Generally, MFR ≥2.5 is considered normal, while MFR <2 indicates a reduction.

Quantitative data were averaged for each segment (N = 20 × 17 = 340 segments) and vascular territory (N = 20 × 3 = 60 territories) from data reconstructed with TOF-OSEM and 3D-RAMLA. We first validated intra-observer reproducibility running two quantitative analyses using PMOD software at intervals of 1 month or more. We then compared rest and stress MBF and MFR from TOF-OSEM and 3D-RAMLA.

### Statistics

All statistical analyses were performed using SPSS version 19 for Windows (SPSS Inc., Chicago, IL). Data were expressed as mean ± SD. During the visual assessment, using a 5-point scale system and count profile analyses, the nonparametric Wilcoxon signed-rank test was used to compare TOF-OSEM and 3D-RAMLA images. A Pearson’s product moment correlation coefficient and a standard linear regression analysis were also used to describe the correlation between the absolute quantitative values of the intra-observer validation and the two reconstruction methods (TOF-OSEM and 3D-RAMLA). Agreement between the reconstruction methods was assessed by Bland-Altman plots. MBF and MFR values obtained from two reconstructions were compared using a paired Student’s *t* test. A *P* value <.05 was deemed statistically significant.

## Results

### Visual Assessment

**Figure 1:**
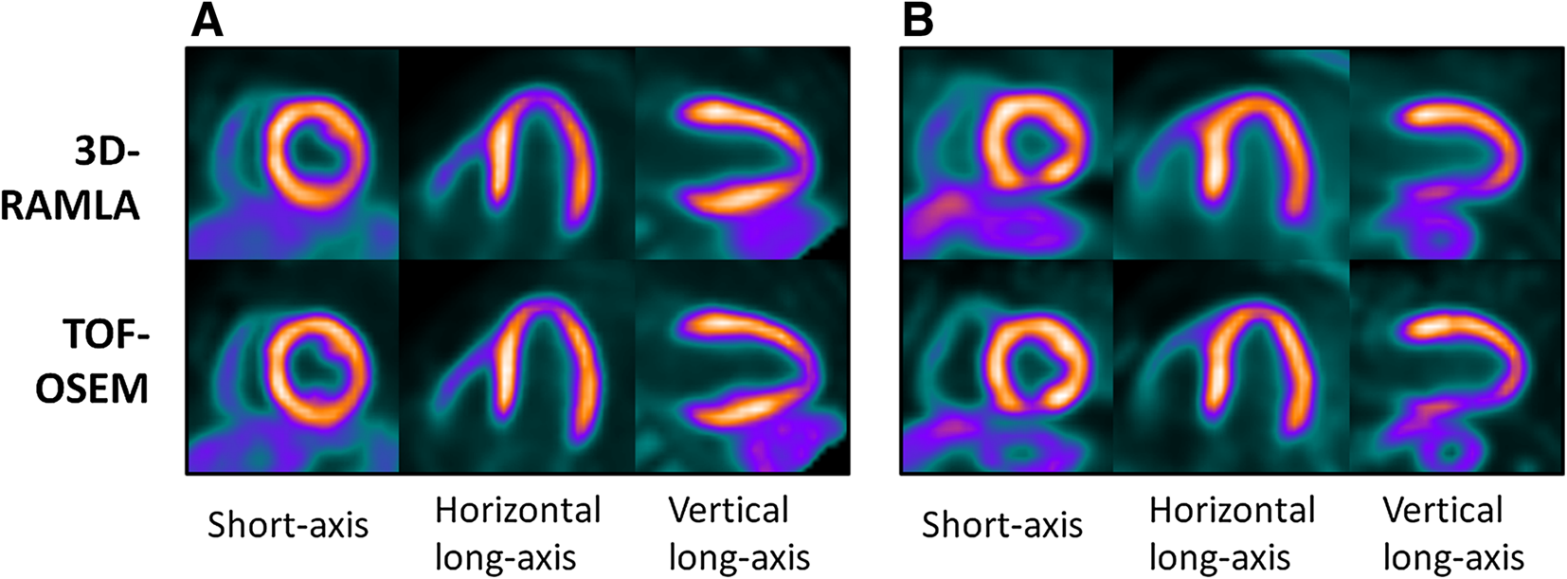
Comparison of images between TOF-OSEM and 3D-RAMLA. (**A**) A representative case of a 53-year-old normal, female volunteer. (**B**) A case with three-vessel coronary artery disease. There was a severe decrease of uptake in the inferior wall

Two sets of images from TOF-OSEM and 3D-RAMLA of a 53-year-old normal female and a case with three-vessel coronary stenosis are shown in Figure 1. The depiction of the myocardium and the discrimination from the heart to the liver was improved by the TOF method. The uptake in the lateral wall was higher with TOF-OSEM than that with 3D-RAMLA. Visual assessment using a 5-point scoring scale system for all subjects revealed that the scores obtained using TOF-OSEM and 3D-RAMLA were approximately similar for all segments, except for those in LAD territory under stress conditions (TOF-OSEM: 2.95 vs 3D-RAMLA: 2.65, *P* = .03). There were no statistical differences of the scores between reconstructions in normal volunteers. The CAD patients had higher scores with TOF-OSEM reconstruction than those with 3D-RAMLA only in LAD territory under stress conditions (TOF-OSEM: 4.31 vs 3D-RAMLA: 3.92, *P* = .059). According to CAG results as a gold standard, sensitivity/specificity with TOF-OSEM and with 3D-RAMLA was 82.1/81.8% and 67.9/81.8% for 13 CAD patients, respectively. This relative high sensitivity with TOF-OSEM was due to accurate detection of CAD in LAD and LCX territories.

### Count Profile Analysis

**Figure 2:**
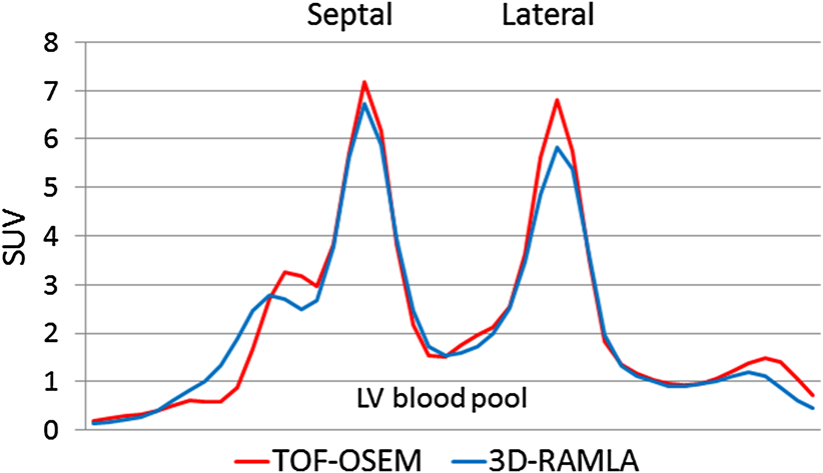
A representative case of the count profile curves. TOF increased the maximum count of the septal and lateral walls. The increase in the count was more evident in the lateral wall. *LV*, left ventricular; *SUV*, standardized uptake value

**Figure 3:**
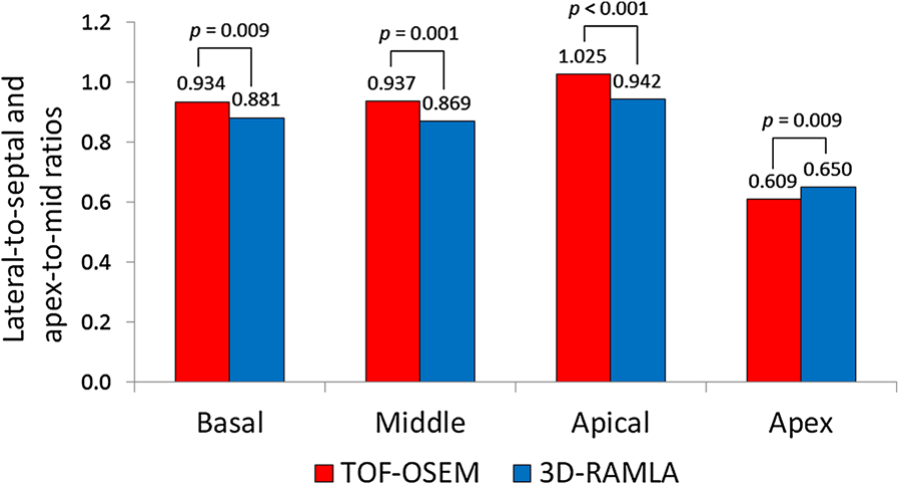
Results of the count profile analysis. Accumulation of ^13^N-ammonia at the lateral walls increased after TOF use. Thus, lateral-to-septal ratio was approximately 1. Moreover, apical thinning was emphasized in TOF images

A representative count profile curve of the mid-short axis under rest conditions is seen in Figure 2. The maximum counts of septal and lateral walls using TOF-OSEM were higher than those using 3D-RAMLA. This count increase was more evident in the lateral wall. Figure 3 represents the lateral-to-septal and apex-to-mid ratios. TOF increased the lateral uptake of ^13^N-ammonia, and brought the lateral-to-septal ratio to 1. In addition, apical thinning was emphasized on TOF images. The myocardial contrasts to blood-pool on the TOF-OSEM and 3D-RAMLA images are shown in Table 2. TOF significantly improved myocardial contrast at all levels of the short axis.

**Table 2:** The myocardial contrast to the blood-pool from TOF-OSEM and 3D-RAMLA reconstructions

N = 20	Basal		Middle		Apical	
	Septal	Lateral	Septal	Lateral	Septal	Lateral
3D-RAMLA	0.723	0.690	0.643	0.599	0.513	0.489
TOF-OSEM	0.743	0.727	0.673	0.653	0.558	0.565
*P* value	.012	.001	<.001	<.001	.001	<.001

### Absolute Quantification of MBF

The intra-observer correlation for MBF and MFR was excellent using both TOF-OSEM and 3D-RAMLA methods, as shown in Table 3. Correlation coefficient of the intra-observer correlation was ranged from 0.964 to 0.991 in normal volunteers (N = 119 segments), while CAD patients (N = 221 segments) showed the values between 0.940 and 0.979.

**Table 3:** Intra-observer comparisons

Segments	3D-RAMLA (mean ± SD)				TOF-OSEM (mean ± SD)			
(N = 340)	First	Second	Difference	*r*	First	Second	Difference	*r*
Rest MBF	0.839 ± 0.184	0.834 ± 0.189	0.033 ± 0.037	0.965	0.964 ± 0.204	0.947 ± 0.203	0.043 ± 0.044	0.956
Stress MBF	2.068 ± 0.715	2.048 ± 0.731	0.096 ± 0.086	0.985	2.160 ± 0.697	2.138 ± 0.702	0.102 ± 0.103	0.979
MFR	2.47 ± 0.75	2.46 ± 0.75	0.12 ± 0.12	0.974	2.24 ± 0.58	2.26 ± 0.57	0.12 ± 0.11	0.960

Territories	3D-RAMLA (mean ± SD)				TOF-OSEM (mean ± SD)			
(N = 60)	First	Second	Difference	*r*	First	Second	Difference	*r*
Rest MBF	0.840 ± 0.158	0.831 ± 0.159	0.024 ± 0.025	0.978	0.959 ± 0.169	0.939 ± 0.162	0.031 ± 0.025	0.979
Stress MBF	2.095 ± 0.665	2.065 ± 0.671	0.066 ± 0.046	0.994	2.159 ± 0.617	2.135 ± 0.617	0.072 ± 0.063	0.989
MFR	2.50 ± 0.71	2.48 ± 0.70	0.09 ± 0.08	0.986	2.25 ± 0.50	2.26 ± 0.49	0.09 ± 0.07	0.975

The intra-observer correlation for MBF and MFR was excellent for both TOF-OSEM and 3D-RAMLA reconstructions (*r* = 0.956 to 0.994). *Difference*, mean of the absolute difference between comparisons; *r*, correlation coefficient

**Figure 4:**
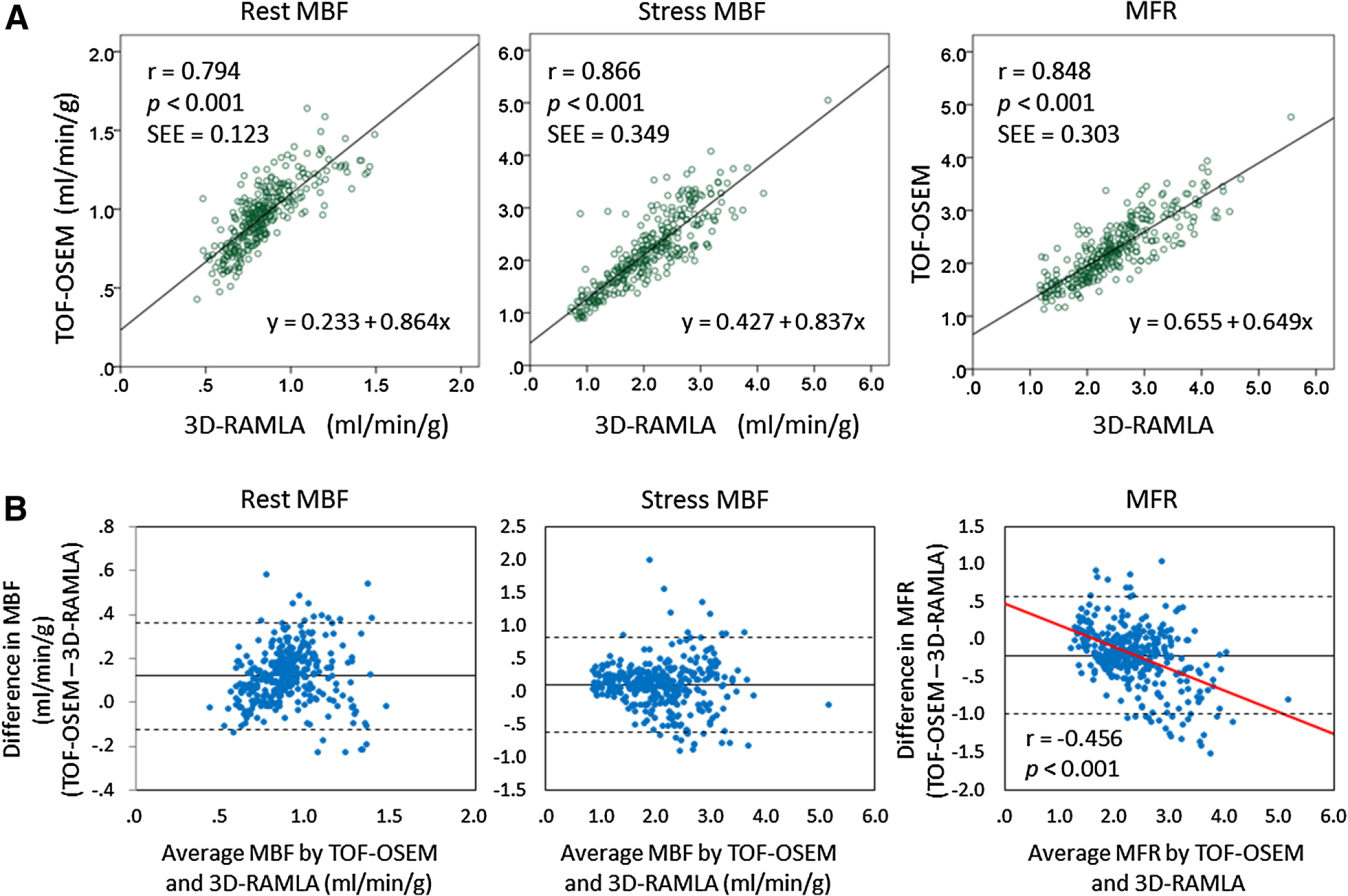
Segmental linear regression analyses and Bland-Altman plots of the differences between TOF-OSEM and 3D-RAMLA. (**A**) Segmental linear regression analyses of quantitative values. (**B**) Bland-Altman plots of the differences in the values of TOF-OSEM and 3D-RAMLA versus the corresponding average values. The *solid lines*, *broken lines*, and *red line* represent the mean biases, the 95% confidence intervals, and the correlation line, respectively

**Figure 5:**
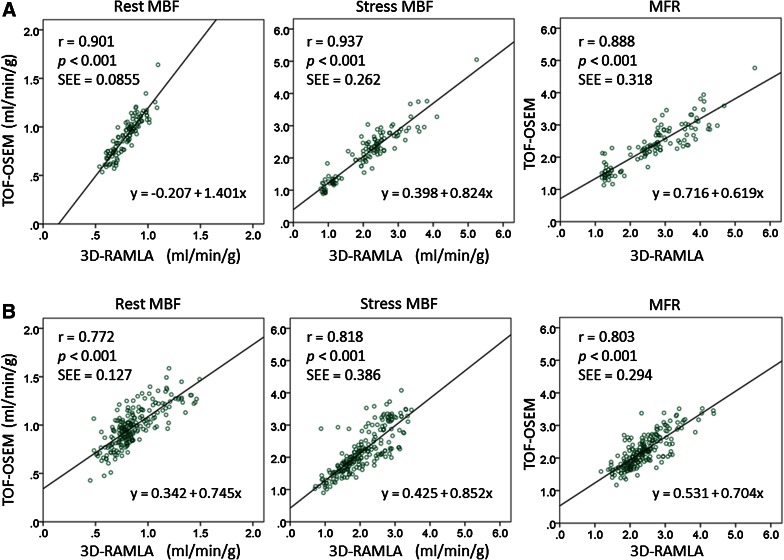
Segmental linear regression analyses of MBF and MFR between TOF-OSEM and 3D-RAMLA. (**A**) Normal volunteers (N = 119 segments) (**B**) CAD patients (N = 221 segments)

**Figure 6:**
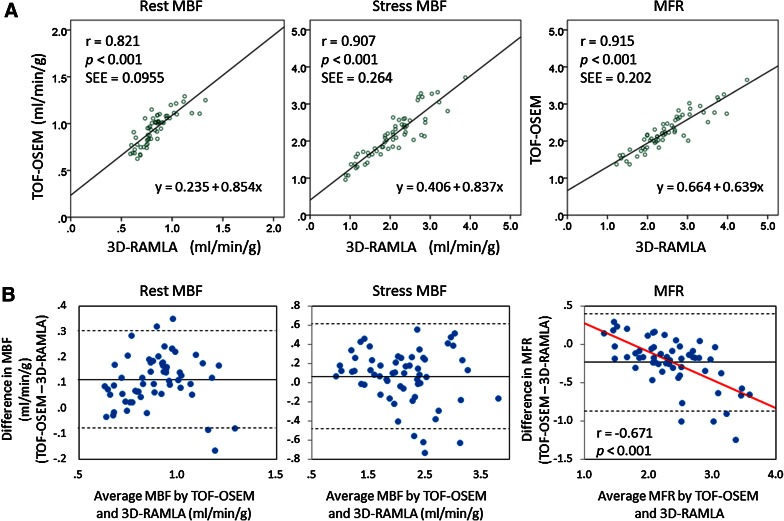
Territorial linear regression analyses and Bland-Altman plots of the differences between TOF-OSEM and 3D-RAMLA. (**A**) Territorial linear regression analyses of MBF and MFR. (**B**) Bland-Altman plots of the differences in the values of TOF-OSEM and 3D-RAMLA vs the corresponding average values. The *solid lines*, *broken lines*, and *red line* represent the mean biases, the 95% confidence intervals, and the correlation line, respectively

Segmental linear regression analyses and Bland-Altman plots are shown in Figure 4. Segmental parameters from TOF-OSEM were well correlated with those from 3D-RAMLA under each condition. Bland-Altman analysis demonstrated that, rest MBF values from TOF-OSEM exceeded those from 3D-RAMLA by +0.119 (95% CI −0.126 to +0.364), stress MBF values from TOF-OSEM exceeded those from 3D-RAMLA by +0.090 (95% CI −0.630 to +0.811), and the MFR from TOF-OSEM was lower than that from 3D-RAMLA by −0.210 (95% CI −0.994 to +0.574). Furthermore, there was a significant negative correlation between the average and difference of MFR between the two methods (*r* = −0.456, *P* < .001), shown in the right column of Figure 4B. Segmental linear regression analyses in normal volunteers and CAD patients are shown in Figure 5. Territorial linear regression analyses and Bland-Altman plots were very similar to the segmental analyses (Figure 6). There was again a significant negative correlation between the average and difference of MFR between the two reconstructions (*r* = −0.671, *P* < .001), shown in the right column of Figure 6B. That is, MFR with TOF-OSEM was lower than that with 3D-RAMLA in normal myocardium.

The differences between the quantitative values from TOF-OSEM and 3D-RAMLA are shown in Table 4. Both segmental rest and stress MBF values from TOF-OSEM were higher than those from 3D-RAMLA (rest MBF: 0.955 ± 0.201 vs 0.836 ± 0.185, *P* < .001 and stress MBF: 2.149 ± 0.697 vs 2.058 ± 0.721, *P* < .001). Differentiation of segmental stress MBF by the TOF method was higher in CAD patients (6.0%) than that in normal volunteers (1.5%). Furthermore, differences in MBF between reconstructions were enhanced under rest conditions. As such, segmental MFR via TOF-OSEM was lower than that using 3D-RAMLA (2.25 ± 0.57 vs 2.46 ± 0.75, *P* < .001). No remarkable differences were observed between segmental and territorial results. However, in the territorial analysis, we did see a small increase in the stress MBF using TOF-OSEM, but the increase was not statistically significant. Additionally, almost all values of coefficient of variances (CV) for TOF-OSEM were lower than those for 3D-RAMLA.

**Table 4:** Differentiation of MBF and MFR by the TOF method

All subjects (N = 20)	Segments (N = 340)			Territories (N = 60)		
	Rest MBF	Stress MBF	MFR	Rest MBF	Stress MBF	MFR
3D-RAMLA	0.836 ± 0.185 (0.221)	2.058 ± 0.721 (0.350)	2.46 ± 0.75 (0.303)	0.836 ± 0.159 (0.244)	2.080 ± 0.673 (0.354)	2.49 ± 0.71 (0.324)
TOF-OSEM	0.955 ± 0.201 (0.211)	2.149 ± 0.697 (0.324)	2.25 ± 0.57 (0.253)	0.949 ± 0.166 (0.232)	2.147 ± 0.621 (0.325)	2.26 ± 0.50 (0.273)
Differentiation by TOF	+14.2%	+4.4%	−8.5%	+13.5%	+3.2%	−9.2%
*P* value	<.001	<.001	<.001	<.001	n.s.	<.001

Volunteers (N = 7)	Rest MBF	Stress MBF	MFR	Rest MBF	Stress MBF	MFR
3D-RAMLA	0.795 ± 0.127 (0.159)	2.094 ± 0.850 (0.404)	2.63 ± 0.99 (0.373)	0.795 ± 0.115 (0.141)	2.108 ± 0.831 (0.385)	2.65 ± 1.00 (0.367)
TOF-OSEM	0.906 ± 0.197 (0.216)	2.125 ± 0.748 (0.350)	2.35 ± 0.69 (0.292)	0.904 ± 0.178 (0.192)	2.112 ± 0.717 (0.331)	2.33 ± 0.66 (0.276)
Differentiation by TOF	+14.0%	+1.5%	−10.6%	+13.7%	+0.2%	−12.1%
*P* value	<.001	n.s.	<.001	<.001	n.s.	.002

CAD patients (N = 13)	Rest MBF	Stress MBF	MFR	Rest MBF	Stress MBF	MFR
3D-RAMLA	0.859 ± 0.207 (0.240)	2.039 ± 0.642 (0.314)	2.37 ± 0.56 (0.236)	0.858 ± 0.176 (0.203)	2.065 ± 0.581 (0.278)	2.40 ± 0.49 (0.200)
TOF-OSEM	0.982 ± 0.199 (0.203)	2.162 ± 0.669 (0.309)	2.20 ± 0.49 (0.223)	0.973 ± 0.156 (0.158)	2.166 ± 0.571 (0.260)	2.22 ± 0.39 (0.172)
Differentiation by TOF	+14.3%	+6.0%	−7.2%	+13.4%	+4.9%	−7.5%
*P* value	<.001	<.001	<.001	<.001	.048	<.001

Quantitative data are expressed as mean ± SD (CV). Both the segmental rest and stress MBF values from TOF-OSEM were higher than those from 3D-RAMLA, especially under rest conditions; as such, the segmental MFR via TOF-OSEM reconstruction was lower. Territorial quantitative analyses were almost similar to segmental analyses. However, an observed increase in the stress MBF from TOF-OSEM was not statistically significant. Almost all values of coefficient of variances for TOF-OSEM were lower than those for 3D-RAMLA; *n.s.*, not statistically significant; *CV*, coefficient of variance

**Figure 7:**
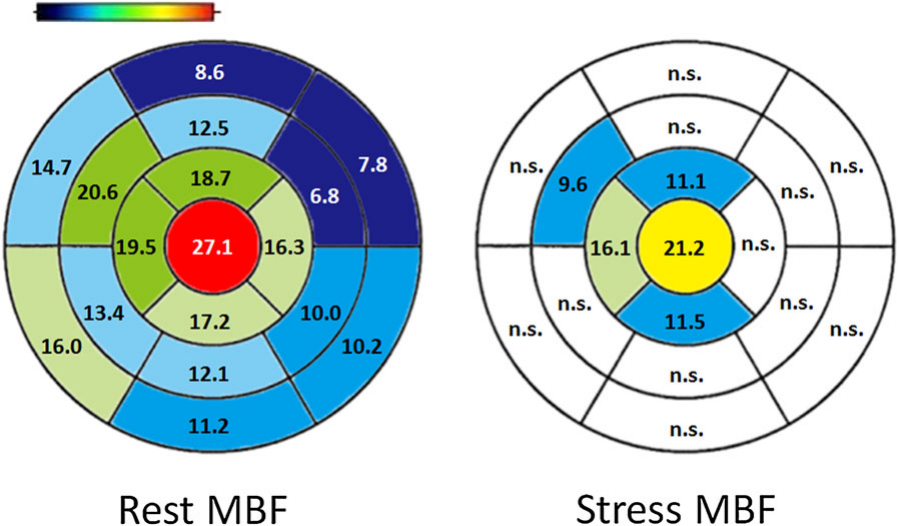
Percent increase in each segment. The rest myocardial perfusion of all segments was significantly larger using TOF-OSEM than that using 3D-RAMLA. Apical and septal segments showed larger values than basal and lateral ones, respectively. On the other hand, only apical segments showed a significantly higher perfusion by TOF use under the stress conditions; *n.s.*, not statistically significant

We also investigated the alterations in MBF of each segment (Figure 7). The rest myocardial perfusion in all segments using TOF-OSEM was significantly larger than that using 3D-RAMLA. Apical and septal segments showed larger values than basal and lateral ones, respectively. On the other hand, only apical segments showed significantly higher perfusion using the TOF method under the stress condition.

## Discussion

### Qualitative Analysis

Both visual and count profile analyses revealed an emphasis of the apical thinning on TOF images in comparison with that on 3D-RAMLA images. In general, the apical wall is relatively thin and yields a lower count during nuclear imaging. Thus, TOF could improve the image quality of the myocardium. As apical thinning is more evident in PET than in SPECT, clinicians should be careful, while assessing apical perfusion imaging with PET, particularly with TOF-PET.

The myocardial retention of ^13^N-ammonia has been reported to be heterogeneous, with the lateral uptake being about 10% lower than that in the other walls, even in healthy subjects.^10^ Main cause of lower perfusion in the lateral wall may be the misregistration between PET and CT images due to respiratory and/or cardiac motion.^19^ In the present study, the relative low count in the lateral wall became unremarkable on TOF-OSEM images. Because the raw data and the alignment of PET and CT images were same between TOF-OSEM and 3D-RAMLA images, we have to consider other mechanisms besides misregistration between PET and CT images. As such, we hypothesized the following mechanism: since the magnitude of the wall motion alone can influence the myocardial accumulation of the tracer, the lateral count in the free wall would likely be lower than the septal one; TOF is able to more precisely detect intrinsic signals and, therefore, may be able to ease this deterioration of the lateral counts. In addition, the signal-to-noise ratio (myocardial contrast to blood-pool) at each level of the short axis using TOF-OSEM was better than that using 3D-RAMLA. Thus, TOF improved the reproducibility of the visual interpretation.

Although specificity to detect CAD based on comparison of rest and stress images was equivalent between TOF-OSEM and 3D-RAMLA, TOF-OSEM showed greater sensitivity than 3D-RAMLA as a consequence of accurate depiction of apical thinning as well as lateral wall.

### Quantitative Analysis

Traditionally, quantitative analysis of myocardial perfusion using PET was adopted using two-dimensional acquisition with a rotating transmission rod source and a filtered back-projection (FBP). Currently, hybrid PET/CT scanners and iterative reconstruction methods have also been widely used. Schepis et al reported that the quantitative measurements of MBF with 3-D PET reconstructed using OSEM were in excellent agreement with those obtained using the 2-D technique and FBP.^20^ Furthermore, a low-dose CT attenuation correction without ECG gating was demonstrated to provide reliable and repeatable global and regional MBF results in comparison of rotating ^68^Ge source for the attenuation correction.^21^

To our knowledge, the current study is the first to investigate the effects of the TOF acquisition technique for the absolute quantification of MBF with ^13^N-ammonia. A phantom and clinical oncological PET studies have both shown that TOF increased the gamma counts in lower count areas and small lesions, respectively.^11^,^12^ The present study results correspond to these findings, showing an improvement in the detection of counts in the lower count area. As a result, MFR decreased by about 10% in our TOF data. Moreover, TOF-OSEM showed lower CV of MBF and MFR, probably due to high signal-to-noise ratio. Taken together, we hypothesize that TOF and non-TOF PET/CT system should have different thresholds of MBF and MFR. Following TOF, differences in MBF in the apical segments were significantly higher perfusion per gram of the myocardium. These results were because TOF was able to detect more counts in the thin myocardium. Furthermore, as TOF increased MBF in low count areas, conventional PET scanners may actually overestimate scar tissue and/or ischemia; alternatively, TOF-PET scanners may underestimate them.

## Limitations

The results of this study were dependent on the specificity of the scanner, acquisition protocols, reconstruction methods, and quantitative software. Moreover, TOF methods differ by vendor; only Philips hardware and PMOD software were used in this study. Quantitative values obtained using other instruments or at other institutions should be compared with care.

## New Knowledge Gained

TOF improves the image quality and the reproducibility of the visual interpretation. Quantitatively, TOF increases MBF, especially under relatively low perfusion conditions.

## Conclusions

The current study revealed that TOF influenced the image quality and quantitative values of myocardial perfusion ^13^N-ammonia PET. The TOF method improved visualization of the myocardium and the differentiation between the heart and the liver. TOF increased the lateral counts in normal perfusion subjects and improved the signal-to-noise ratio. However, because apical thinning was emphasized by the TOF method, the apex should be scored carefully. During quantitative analysis, we also found that TOF reconstruction increased MBF, especially under relatively low perfusion conditions.
